# Comments on the Report of the Committee on M. Pasteur's Treatment of Rabies and Hydrophobia

**Published:** 1888-06

**Authors:** 


*Comments on the Report of the Committee on M. Pasteur's Treatment of Rabies and Hydrophobia.* By Surgeon-General C. A. GORDON, M.D. Ed. Pp. 96. London: BAILLIÈRE & Co. 1888.

We had occasion last year to comment unfavourably on a work published by our author on *Inoculation for Rabies and Hydrophobia.* In that work, he says in the preface to the one before us, "he endeavoured to summarise as much of the literature of the subject as came within his knowledge." We then pointed out that this literature was of the nature found on the table of the reading-room of a general London club, and was conspicuous for its frequent references to the *Pall Mall Gazette* and the *Daily News;* and we ventured to advise our readers that the work could not be looked upon as either serious or scientific. The same criticism, though less severely, applies to the work before us. The author exhibits a strange predilection for popular as distinguished from scientific sources for his truths; and he shows a uniform and almost pitiable inability to grasp the main features of the profound problem he attacks. It is impossible to speak without respect of one who has attained to the rank and repute of Surgeon-General Gordon; in his case (as in that of so many others) it would be as well if "on the retired list" could be applied to science as well as to soldiering.

